# Clinicopathological and molecular characterization of a case classified by DNA‑methylation profiling as “CNS embryonal tumor with *BRD4–LEUTX* fusion”

**DOI:** 10.1186/s40478-023-01549-2

**Published:** 2023-03-18

**Authors:** Laetitia Lebrun, Sacha Allard-Demoustiez, Nathalie Gilis, Claude Van Campenhout, Marine Rodesch, Celine Roman, Pierluigi Calò, Valentina Lolli, Philippe David, Christophe Fricx, Olivier De Witte, Fabienne Escande, Claude-Alain Maurage, Isabelle Salmon

**Affiliations:** 1 Department of Pathology, Université Libre de Bruxelles (ULB), Hôpital Universitaire de Bruxelles (HUB), CUB Hôpital Erasme, Erasme University Hospital, Brussels, Belgium; 2 Department of Neurosurgery, Université Libre de Bruxelles (ULB), Hôpital Universitaire de Bruxelles (HUB), CUB Hôpital Erasme, Erasme University Hospital, Brussels, Belgium; 3 Department of Pediatric, Université Libre de Bruxelles (ULB), Hôpital Universitaire de Bruxelles (HUB), CUB Hôpital Erasme, Erasme University Hospital, Brussels, Belgium; 4 Université Libre de Bruxelles (ULB), Hôpital Universitaire de Bruxelles (HUB), CUB Hôpital Universitaire Des Enfants Reine Fabiola, Brussels, Belgium; 5 Department of Radiology, Université Libre de Bruxelles (ULB), Hôpital Universitaire de Bruxelles (HUB), CUB Hôpital Erasme, Erasme University Hospital, Brussels, Belgium; 6 Service de Biochimie et Biologie Moléculaire, Pole Pathologie Biologie, CHU Lille, Lille, France; 7 UFR3S - Laboratoire d’Histologie, Univ. Lille, 59000 Lille, France; 8 Inserm, U1172 - Lille Neuroscience & Cognition, 59000 Lille, France; 9 Institut de Pathologie, CHU Lille, 59000 Lille, France; 10 DIAPath, Center for Microscopy and Molecular Imaging (CMMI), ULB, Gosselies, Belgium

**Keywords:** Brain tumor, CNS embryonal tumor, DNA methylation, H3K27me3 protein expression, BRD4, LEUTX

## Abstract

Central Nervous System (CNS) embryonal tumors represent a heterogeneous group of highly aggressive tumors occurring preferentially in children but also described in adolescents and adults. In 2021, the CNS World Health Organization (WHO) classification drastically changed the diagnosis of the other CNS embryonal tumors including new histo-molecular tumor types. Here, we report a pediatric case of a novel tumor type among the other CNS embryonal tumors classified within the methylation class “CNS Embryonal Tumor with *BRD4–LEUTX* Fusion”. The patient was a 4-year girl with no previous history of disease. For a few weeks, she suffered from headaches, vomiting and mild fever associated with increasing asthenia and loss of weight leading to a global deterioration of health. MRI brain examination revealed a large, grossly well-circumscribed tumoral mass lesion located in the left parietal lobe, contralateral hydrocephalus and midline shift. Microscopic examination showed a highly cellular tumor with a polymorphic aspect. The majority of the tumor harbored neuroectodermal features composed of small cells with scant cytoplasm and hyperchromatic nuclei associated with small “medulloblastoma-like” cells characterized by syncytial arrangement and focally a streaming pattern. Tumor cells were diffusely positive for Synaptophysin, CD56, INI1 and SMARCA4 associated with negativity for GFAP, OLIG-2, EMA, BCOR, LIN28A and MIC-2. Additional IHC features included p53 protein expression in more than 10% of the tumor’s cells and very interestingly, loss of H3K27me3 expression. The Heidelberg DNA-methylation classifier classified this case as “CNS Embryonal Tumor with BRD4:LEUTX Fusion”. RNA-sequencing analyses confirmed the *BRD4 (exon 13)–LEUTX (exon 2)* fusion with no other molecular alterations found by DNA sequencing. Our case report confirmed that a new subgroup of CNS embryonal tumor with high aggressive potential, loss of H3K27me3 protein expression, *BRDA4–LEUTX* fusion, named “Embryonal CNS tumor with *BRD4–LEUTX* fusion”*,* has to be considered into the new CNS WHO classification.

## Introduction

Central Nervous System (CNS) embryonal tumors represent a heterogeneous group of highly aggressive tumors occurring preferentially in children but also described in adolescents and adults [17]. Among them, the diagnosis of medulloblastomas using integration of histopathological and molecular features is exemplified [18]. Nevertheless, for the other CNS embryonal tumors, we are at the first step of the emergence of this histo-molecular diagnostic approach. This has been underlined by Sturm et al*.* who revealed in 2016 new molecular entities among these poorly differentiated embryonal tumors [17]. In 2021, the CNS World Health Organization (WHO) classification drastically changed the diagnosis of the other CNS embryonal tumors including new histo-molecular tumor types: CNS neuroblastoma, *FOXR2*-activated, CNS tumor with *BCOR* internal tandem duplication (ITD) and cribriform neuroepithelial tumor [12]. These subtypes are defined by essential criteria combining histopathological, immunohistochemical, molecular as well as epigenetics data [12]. This underlined the heterogeneity of these tumors and the need to further characterization. Here, we report a pediatric case of a novel tumor type among the other CNS embryonal tumors classified within the methylation class (MC) “CNS Embryonal Tumor with BRD4:LEUTX Fusion” (calibrated score (cs): 0.99) which is not yet integrated into the CNS WHO classification.

## Case presentation

**Fig. 1:**
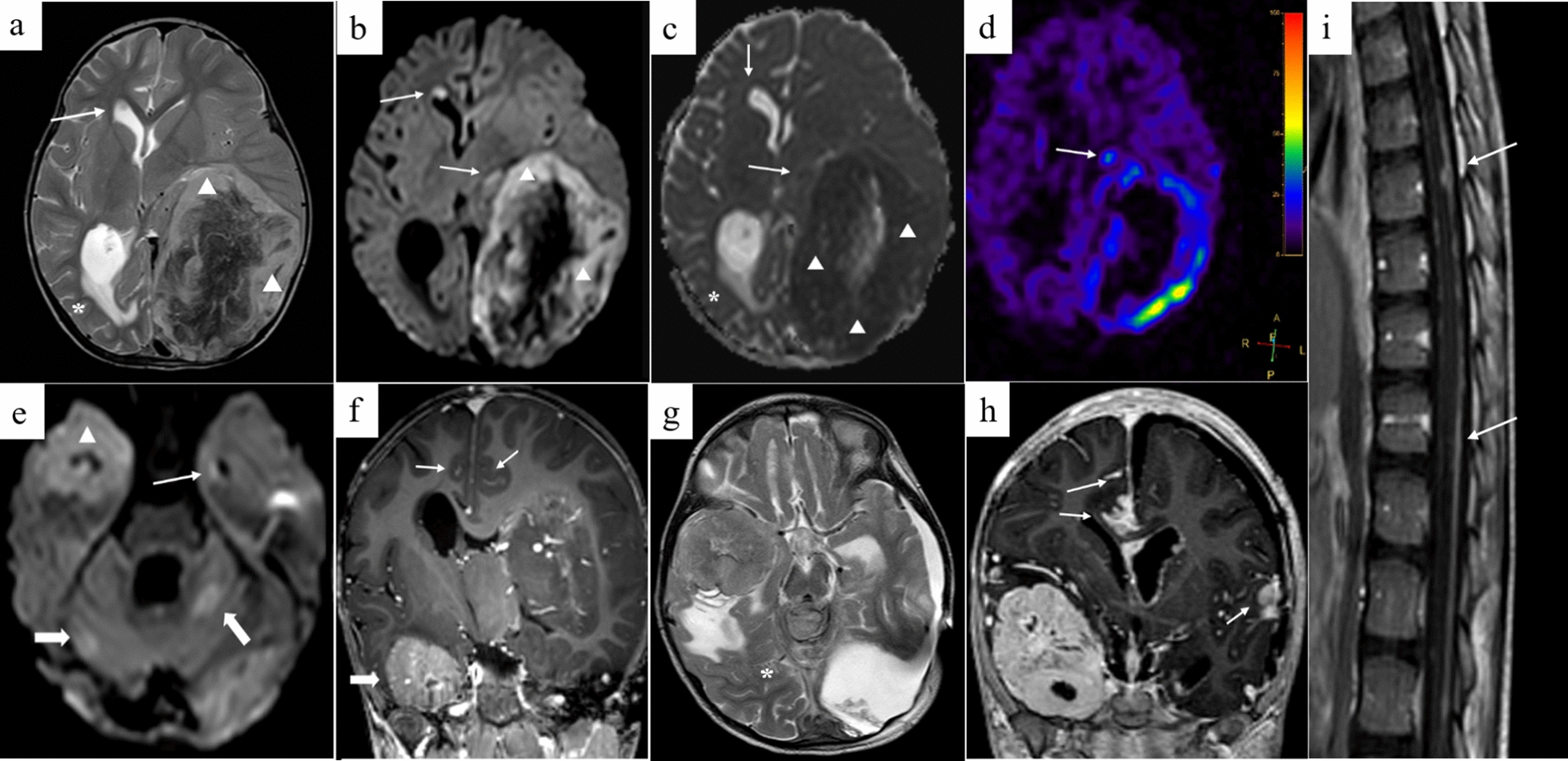
Radiological features. Pre-operative brain MRI **a**–**f** Images revealed a grossly well-marginated tumor mass lesion of approximately 8 × 9 cm located in the left parietal lobe. The lesion exerted severe mass effect on the ipsilateral lateral ventricle and adjacent brain parenchyma, resulting in contralateral active hydrocephalus (*in **a** and **c**) and midline shift. There was only mild peritumoral vasogenic edema. Signal intensity was strikingly heterogenous on all sequences suggesting the presence of core calcifications, necrosis and hemorrhage. The peripheral solid tumoral components displayed significant T2 prolongation (△ in **a**) and restricted diffusion on DW-images **b**, **e** and corresponding ADC map (△ in **c**), indicating high cellularity. ASL perfusion **d** demonstrated markedly increased CBF. Smaller size metastatic intraventricular (arrows in **a**–**e**), leptomeningeal (arrow in **f**) and parenchymal (bold arrows in **e**) lesions were present both supra-and infra-tentorially. The left parietal lobar lesion displayed faint, heterogenous contrast enhancement, whereas the right temporal lobe lesion enhanced avidly. The metastatic intraventricular deposits did not show any contrast uptake. Follow-up brain (**g**, **h**) and spinal cord (**i**) MRI: post contrast coronal T1-weighted (**h**) and sagittal T1-weighted (**i**) images of the brain and spinal cord, four weeks after diagnosis, demonstrated marked interval tumoral increase in size and progression of subependymal and leptomeningeal dissemination (arrows). Surrounding vasogenic edema was also increased (*in **g**). DW: diffusion-weighted; ADC, apparent diffusion coefficient; ASL, arterial spin labeling; CBF, cerebral blood flow

**Fig. 2:**
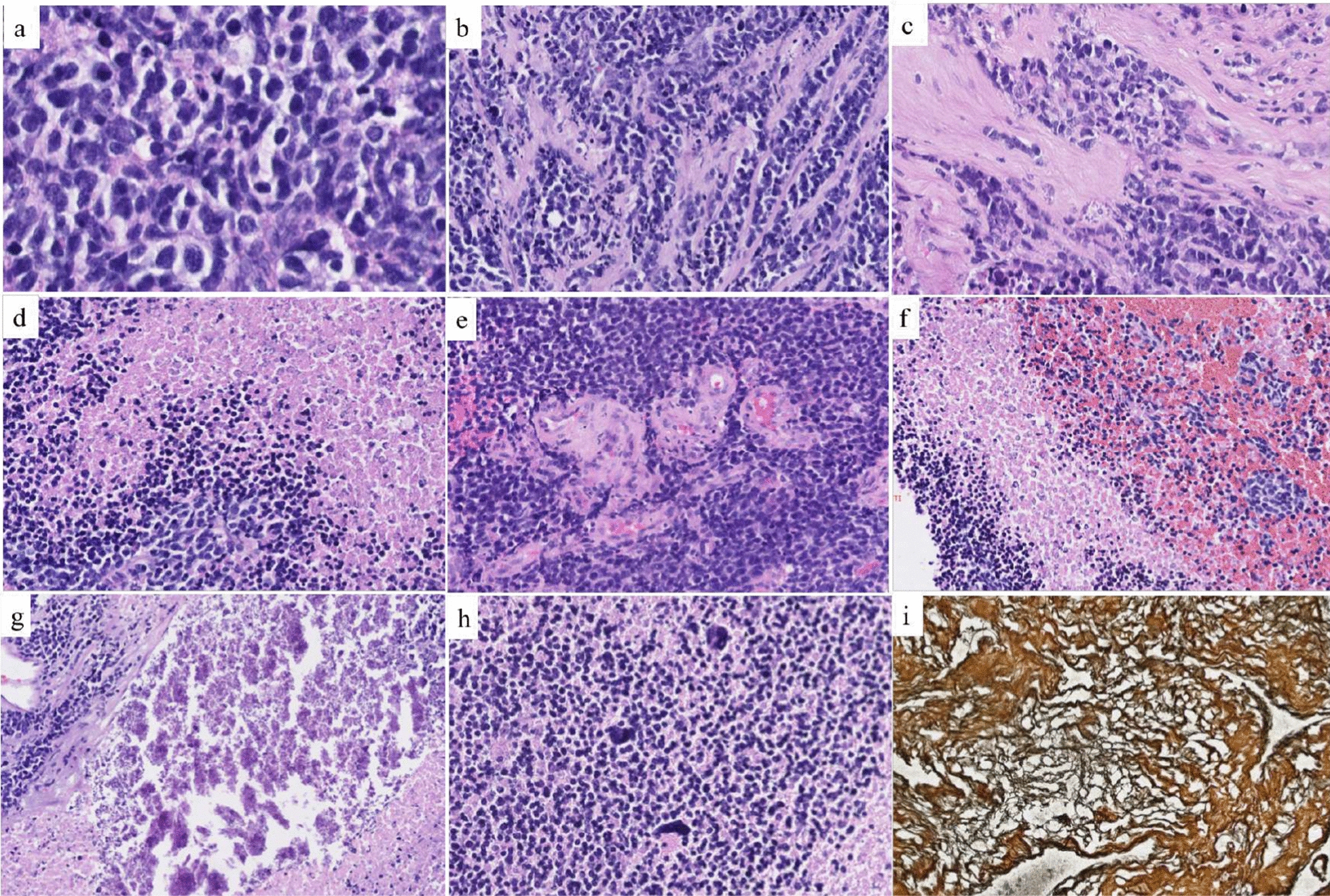
Histopathological features. **a** The tumor was highly cellular, composed of small cells with scant cytoplasm and hyperchromatic nuclei (HE, magnification 400×). **b**, **c** Intratumoral desmoplasia with focally tumoral cells disposed in a streaming pattern (HE, magnification 200×). **d** Tumoral geographical necrosis (HE, magnification 100×). **e**: Microvascular endothelial proliferation (HE, magnification 100×). **f** Intratumoral hemorrhage (HE, magnification 100×). **g** Calcifications (HE, magnification 200×). **h** Presence of numerous “*bizarre* cells” with high nuclear pleomorphism (HE, magnification 200×). **i** Reticulin stain highlighted intense desmoplasia (HE, magnification 200×). HE, Hematoxylin–eosin

**Fig. 3:**
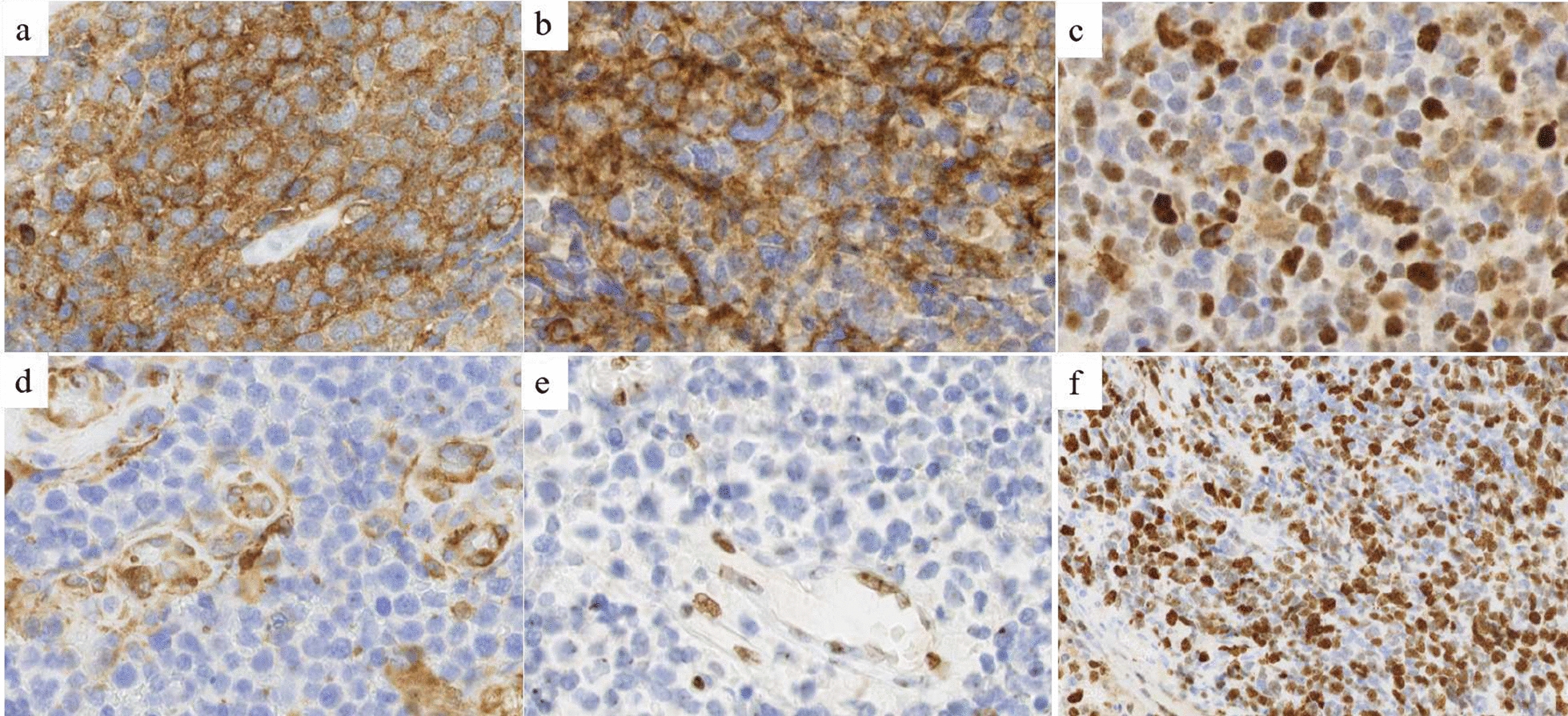
Immunohistochemical features. **a** Diffuse Synaptophysin immunopositivity (magnification 400×). **b** Diffuse CD56 immunopositivity (magnification 400×). **c** Intense nuclear immunopositivity of p53 protein in more than 10% of the tumoral cells (magnification 400×). **d** Loss of Filamine-A expression in tumoral cells with immunopositivity in endothelial cells (magnification 400×). **e** Loss of H3K27me3 expression in tumoral cells with immunopositivity in endothelial cells (magnification 400×). **f** High KI-67 proliferation index with some hotspot of more than 50% of immunopositivity in tumoral cells (magnification 200×)

**Fig. 4:**
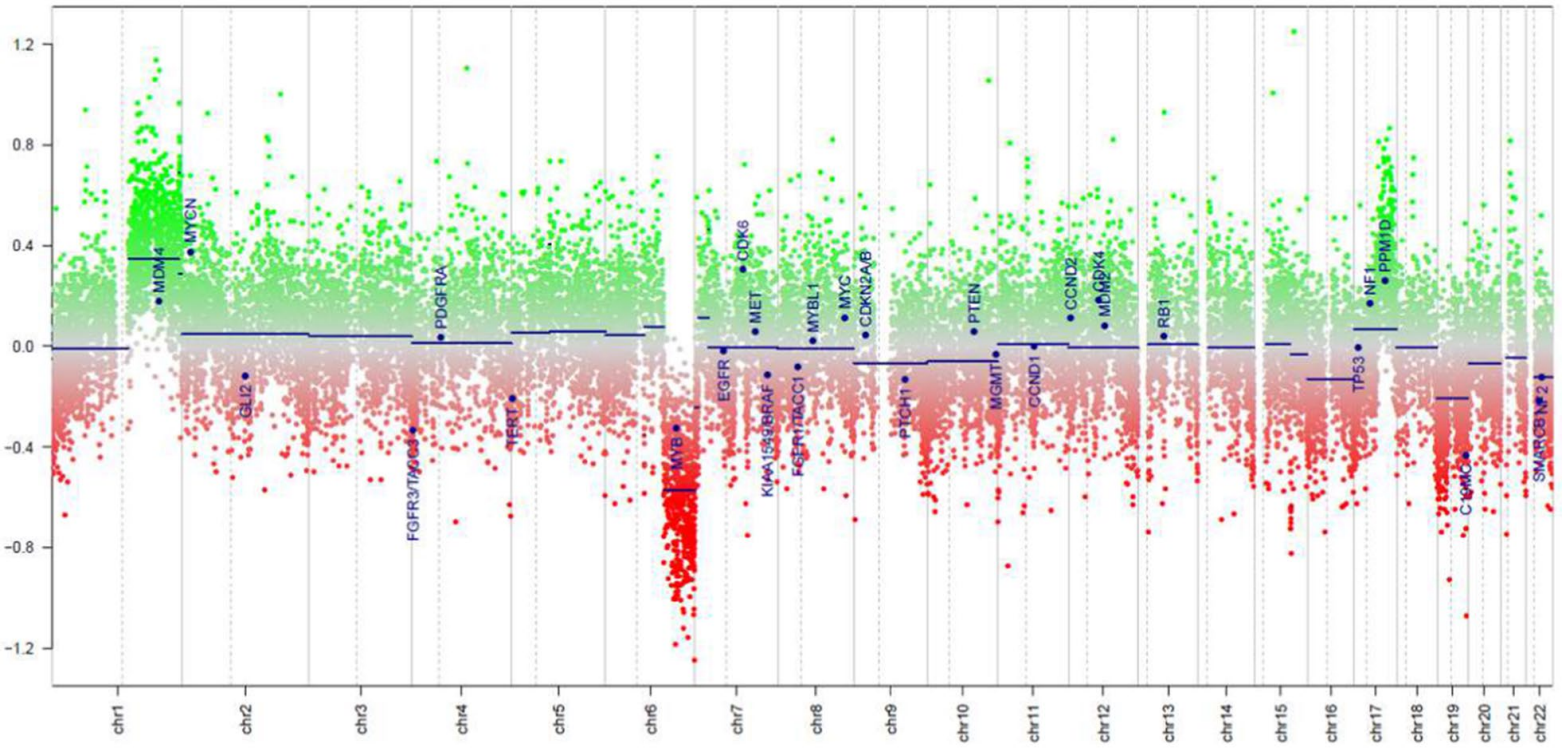
Copy Number Variation (CNV) profile. DNA methylation profiling analyses by the Heidelberg Classifier V12.5 provided CNV profile of the case revealing gain of chromosome 1p and loss of chromosome 6q, provided by https://www.molecularneuropathology.org

The patient was a 4-year girl with no previous history of disease except for intrauterine growth restriction with a birth weight of 1900 g. For a few weeks, she suffered from headaches, vomiting and mild fever associated with increasing asthenia and loss of weight leading to a global deterioration of health. An ophthalmological examination revealed papilledema. A Computed Tomography (CT)-Scan and subsequently a Magnetic Resonance Imaging (MRI) of the brain were therefore obtained, demonstrating a large, grossly well-circumscribed tumoral mass lesion located in the left parietal lobe, contralateral hydrocephalus and midline shift (Fig. 1). There were signs of subependymal and leptomeningeal dissemination, along with several smaller size tumoral lesions both supra- and infratentorial. The proposed radiological diagnosis was that of a metastatic Atypical Teratoid Rhabdoid Tumor (ATRT). Intra-operative aspect confirmed the heterogeneity of the tumor, highly hemorrhagic, with numerous calcifications and necrosis. Microscopic examination showed a highly cellular tumor with a polymorphic aspect (Fig. 2). The majority of the tumor harbored neuroectodermal features composed of small cells with scant cytoplasm and hyperchromatic nuclei associated with small “medulloblastoma-like” cells characterized by syncytial arrangement and focally a streaming pattern. No Homer Wright or Flexner–Wintersteiner rosettes were reported. In some areas the cells harbored moderately bi-, multi-nucleated and pleomorphic nuclei with numerous “*bizarre* cells”. The mitotic activity was high with 5–10 mitoses per 2.3 mm^2^ and KI-67 proliferative index nearing 50%. Numerous apoptotic bodies were observed. Intratumoral desmoplasia was a prominent feature of this tumor as confirmed by Reticulin stain. Microvascular proliferation, extensive geographical necrosis, hemorrhage and calcifications were observed. The tumor was characterized by the lack of ganglion cells, ependymal rosettes, peri-vascular pseudo-rosettes, multilayered structures, nodular formation, cribriform structure, mesenchymal or epithelial differentiation, rhabdoid cells. Immunohistochemistry (IHC) analyses are illustrated in Fig. 3. Tumor cells were diffusely positive for Synaptophysin, CD56, INI1, SMARCA4 and ATRX associated with negativity for GFAP, OLIG-2, EMA, BCOR, LIN28A, EZHIP, MIC-2, Cytokeratin’s, SMA, Desmin and Myogenin. The combination of the morphological and IHC profiles led us to classify this case under “other CNS embryonal tumors, NOS”. Additional IHC features included p53 protein expression in more than 10% of the tumor’s cells and very interestingly, loss of H3K27me3 expression. Regarding molecular analyses, NGS sequencing revealed no mutations in genes commonly involved in CNS tumors such as *ACVR1, IDH1/2, BRAF, H3F3A, HISTH3B/C, TP53, ATRX, TERT, SMARCB1* and *EGFR* genes, extensively described in [5, 11]. NGS testing suggested atypical Chromosome 1q profile with no loss or gain of heterozygoty of chromosome 19. Finally, using the Heidelberg DNA-methylation classifier [3], the case was classified as “CNS Embryonal Tumor with BRD4:LEUTX Fusion” (with a calibrated score (cs) of 0.99). Copy Number Variation (CNV) profile, provided by the online platform https://www.molecularneuropathology.org, suggested gain of chromosome 1q and loss of chromosome 6q (Fig. 4). We confirmed this diagnosis by RNA-sequencing analyses using kit FusionPlex Pan Solid Tumor v.2 (Archer Dx) which revealed *BRD4–LEUTX* fusion. The breakpoint locations are chr19:15355042 and chr19:40275167 for *BRD4* (exon 13; NM_058243.2) and *LEUTX* (exon 2; NM_0001143832.1), respectively. Three weeks after sub-total surgical resection of the left parietal tumor, the residual tumor as well as secondary tumoral locations previously described doubled in size (Fig. 1). Numerous other lesions appeared in supratentorial and infratentorial regions and meningeal dissemination was observed in the spinal cord. Induction chemotherapy with VP16-Carboplatin according to PNET HR + 5 protocol (NCT00936156) was proposed but not feasible due to severe global deterioration. One month after the initial diagnosis, the patient passed away from neurological and clinical complications.

## Discussion and conclusion

*BRD4–LEUTX* fusion is a rare fusion which has only been described in the literature in one infant CNS embryonal tumor (*BRD4* exon 11-*LEUTX* exon 2 fusion) in a comprehensive molecular profiling study of 252 high-risk pediatric cancers [19]. Outside the CNS, this fusion has been reported in one pediatric epithelioid malignant peripheral nerve sheath tumor and an alveolar rhabdomyosarcoma [2, 4]. Even though they share the same molecular alteration, the tumor locations, morphologies and IHC profiles were drastically different between our case and the *BRD4–LEUTX* pediatric sarcoma described by Barresi et al. [2]. Other *LEUTX* gene fusions including *CIC* gene (*CIC–LEUTX* fusion) were already reported in very few cases including one CNS embryonal tumor and an anaplastic pleomorphic astrocytoma [8, 16]. In these studies, no clinico-radiological, histological and DNA methylation profiling were reported. Interestingly, regarding *CIC* gene fusions, *CIC–NUTM1* has been reported in CNS Ewing Sarcoma and represent the majority of CNS CIC-rearranged sarcomas, according to the CNS WHO classification [12, 16]. In 70–80% of cases, *NUTM1* gene is involved in a balanced translocation with *BRD4* gene in NUT carcinoma, which is a poorly differentiated carcinoma occurring mostly in children and adolescents [15]. Therefore, the tumors harboring *BRD4–LEUTX*, *CIC–LEUTX*, *BRD4–NUTM1* and *CIC–NUTM1* fusions seem to be mostly observed in children and young adults. Recent papers suggest that the bromodomain and extraterminal (BET) protein BRD4 could play an important role in the development of brain cancer. BRD4 protein is a chromatin reader protein involved in initial recognition of acetylated histones and plays a key role in epigenetic memory across cell divisions and transcription regulation [13, 20]. LEUTX homeobox protein is normally expressed during early embryogenesis and regulates genes associated with pluripotency and differentiation [10, 19]. Acetylated histones are bound to BRD4 to activate transcription. Therefore, BET bromodomain inhibitors blocking the bind between BRD4 and acetylated histones lead to transcriptional inactivation and have been reported to be promising therapeutic strategy in medulloblastomas and glioblastomas [7, 9]. The potential interchangeable ability of *BRD4* and *CIC* genes regarding fusions with *LEUTX* and *NUTM1* genes could be explained by the fact that both genes i.e. *CIC* and *BRD4* are involved in the transcription regulation and associated with the recruitment of chromatin modifiers such as histone acetyl transferases [16]. Interestingly, in our case, loss of H3K27me3 expression was observed. While widely studied in neuroglial tumors leading to specific entities, the decrease of H3K27me3 expression is less described in CNS embryonal tumors with scarce data available regarding histone modifications alterations in these tumors. Interestingly, loss of H3K27me3 has been described by Alexandrescu et al. in primary intracranial sarcoma, *DICER1*-mutant [1, 14]. Studies reporting effects of targeting therapeutic EZH2 and BET inhibitors in ATRT [9, 21] lead us to propose the hypothesis that the loss of H3K27me3 is linked to the inactivation of BRD4 in this case. YAP1 and Filamine-A IHC, commonly used in the setting of the molecular subgroups of medulloblastomas) [6], were performed and showed loss of expression in tumor cells (Fig. 3), with unknown clinical significance in this tumor type. Finally, regarding morphological specificities, as observed in CNS tumor with *BCOR* ITD, giant, “bizarre” cells with marked cytonuclear pleomorphism associated with geographical necrosis could be helpful to distinguish this tumor type. In addition to these features, large area of intratumoral desmoplasia and calcifications could be specific to this subgroup. These rare cases of CNS embryonal tumor characterized by *BRD4–LEUTX* fusion need more clinical integration associated with prognosis to lead to an integration into the CNS WHO classification.

To conclude, according to the 2021 WHO classification, CNS embryonal tumors are “a work in progress” due to the emergence of a classification based on molecular characteristics [12]. Our case report suggested a new subgroup of CNS embryonal tumor with high aggressive potential, *BRD4–LEUTX* fusion, loss of H3K27me3 protein expression, named “Embryonal CNS tumor with *BRD4–LEUTX* fusion”. This subgroup should be considered into the new CNS WHO classification if more cases reports allow us to better characterized this potential subgroup.

## Data Availability

The datasets used and/or analyzed during the current study are available from the corresponding author upon reasonable request.

## Abbreviations

ITD: Internal tandem duplication
CT: Computed tomography
ATRT: Atypical teratoid rhabdoid tumor
CNS: Central nervous system
CNV: Copy number variations
MRI: Magnetic resonance imaging
NGS: Next generation sequencing
WHO: World Health Organization
IHC: Immunohistochemical
HE: Hematoxylin–eosin
DW: Diffusion-weighted
ADC: Apparent diffusion coefficient
ASL: Arterial spin labeling
CBF: Cerebral blood flow
BET: Bromodomain and extraterminal
